# Supplementary material for: Derivatives of Amaryllidaceae Alkaloid Ambelline as Selective Inhibitors of Hepatic Stage of Plasmodium berghei Infection In Vitro
Source: Pharmaceutics. 2023 Mar 21;15(3):1007. doi: 10.3390/pharmaceutics15031007 (PMC10056443; doi:10.3390/pharmaceutics15031007)
Supplement: Supplementary file 1 [file pharmaceutics-15-01007-s001.zip › Final_Supplementary material_NMR and HRMS.pdf]

Supplementary material for:

Derivatives of Amaryllidaceae Alkaloid Ambelline as Selective Inhibitors of Hepatic Stage of *Plasmodium* Infection *In Vitro*

Kateřina Hradiská Breiterová[*], Aneta Ritomská[1], Diana Fontinha[2], Jana Křoustková[1], Daniela Suchánková[1], Anna Hošťálková[1], Marcela Šafratová[1], Eliška Kohelová[1], Rozálie Peřinová[1], Rudolf Vrabec[1], Denise Francisco[2], Miguel Prudêncio[2] and Lucie Cahlíková[1,*]

[1] Secondary metabolites of plants as potential drugs Research Group, Department of Pharmacognosy and Pharmaceutical Botany, Faculty of Pharmacy, Charles University, Heyrovského 1203, 500 05 Hradec Králové, Czech Republic

[2] Prudêncio Lab, Instituto de Medicina Molecular João Lobo Antunes, Faculdade de Medicina, Universidade de Lisboa, Av. Prof. Egas Moniz, Edf. Egas Moniz, 1649-028 Lisboa, Portugal

*Correspondence: cahlikova@faf.cuni.cz (L.C.),

Table of content

1) MS and NMR spectra of 11-*O*-(3,5-Dimethylbenzoyl)ambelline (**28h**) 3

2) MS and NMR spectra of 11-*O*-(3,5-Dimethoxybenzoyl)ambelline (**28m**) 4

3) MS and NMR spectra of 11-*O*-(3,4,5-Trimethoxybenzoyl)ambelline (**28n**) 6

4) MS and NMR spectra of 11-*O*-(4-Methyl-3-nitrobenzoyl)ambelline (**28r**) 7

5) MS and NMR spectra of 11-*O*-(2-Chloro-4-nitrobenzoyl)ambelline (**28s**) 9

6) MS and NMR spectra of 11-*O*-(4-Chloro-3-nitrobenzoyl)ambelline (**28t**)............................. 10

1) *11-O-(3,5-Dimethylbenzoyl)ambelline (**28h**)*

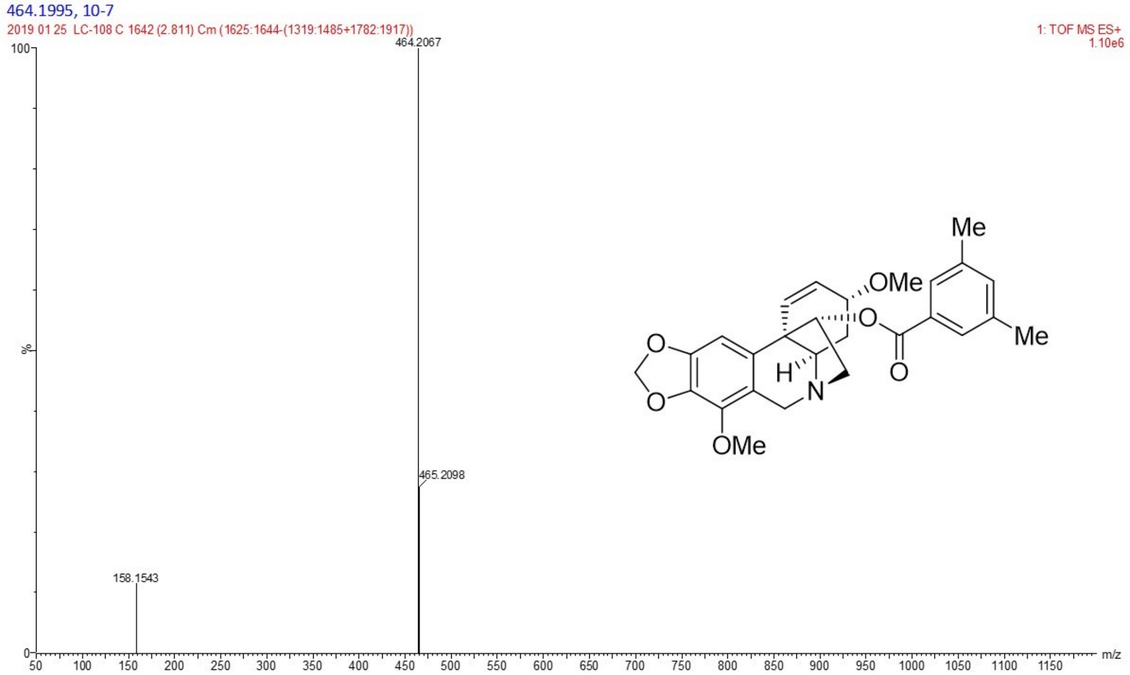

Figure S1. ESI-HRMS spectrum of 11-*O*-(3,5-dimethylbenzoyl)ambelline (**28h**)

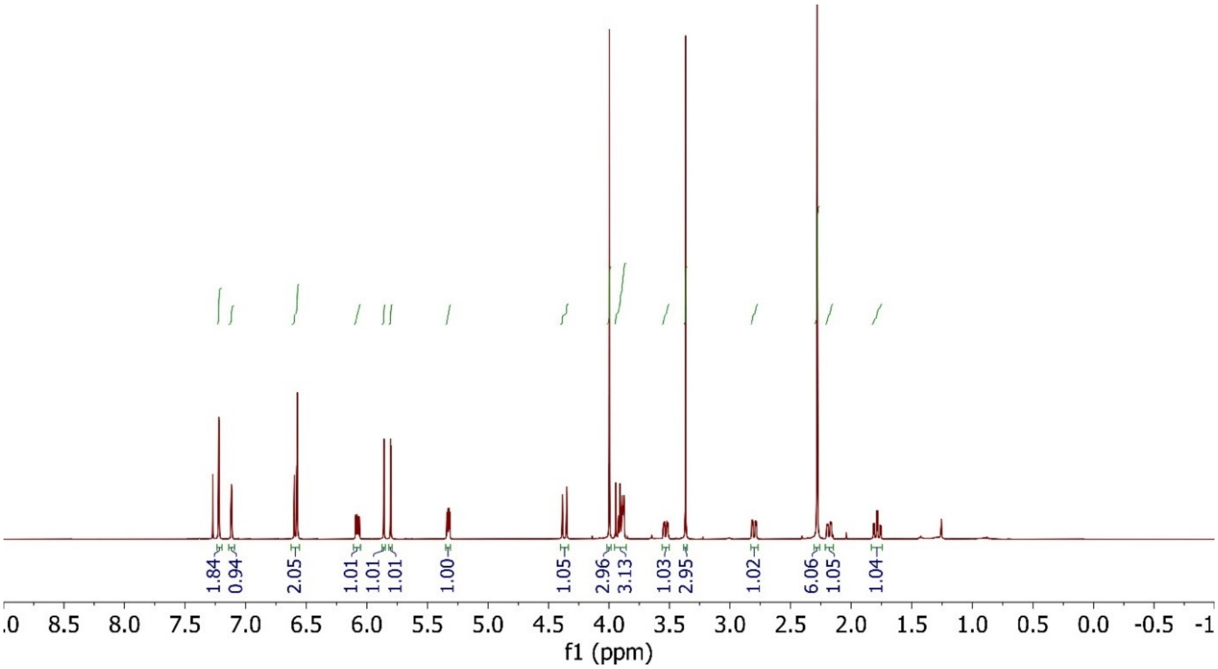

Figure S2. ^{1}H NMR spectrum of 11-*O*-(3,5-dimethylbenzoyl)ambelline (**28h**)

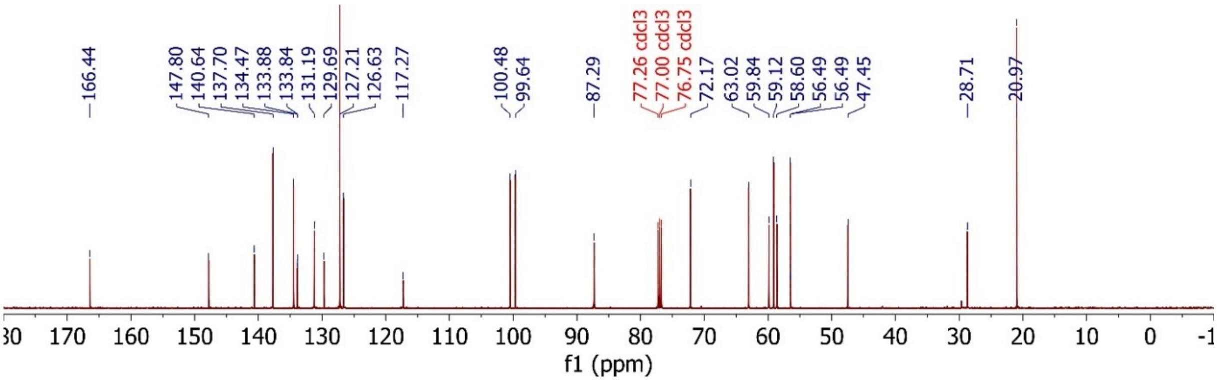

Figure S3. ^{13}C NMR spectrum of 11-*O*-(3,5-dimethylbenzoyl)ambelline (**28h**)

*2) 11-O-(3,5-Dimethoxybenzoyl)ambelline (**28m**)*

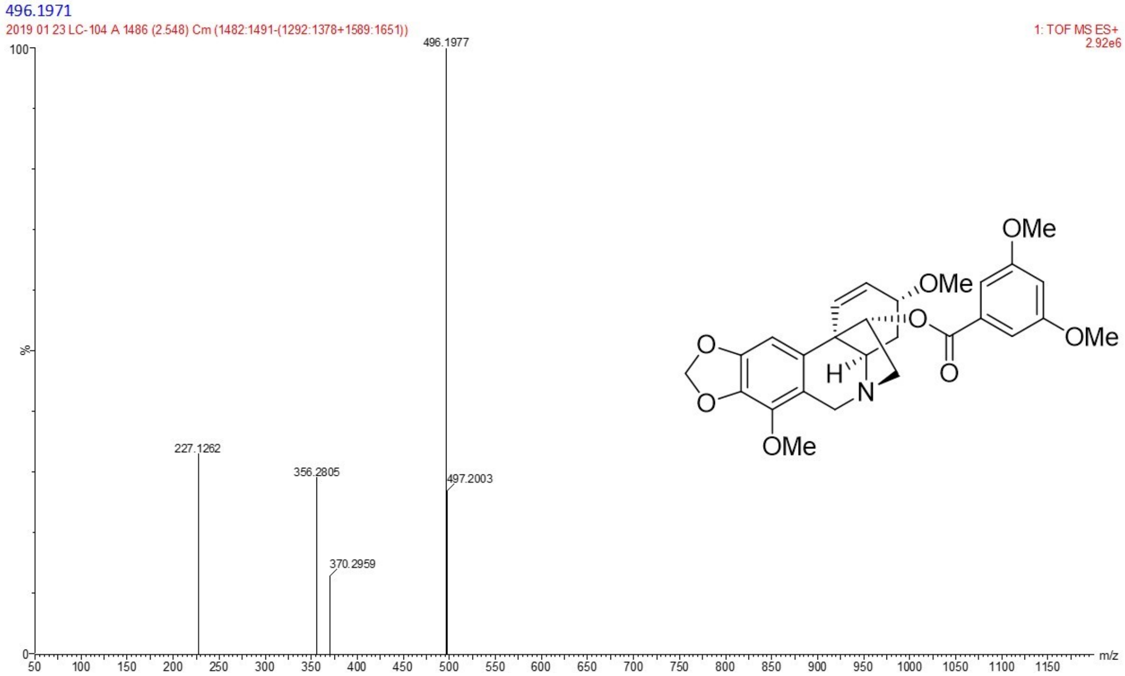

Figure S4. ESI-HRMS spectrum of 11-*O*-(3,5-dimethoxybenzoyl)ambelline (**28m**)

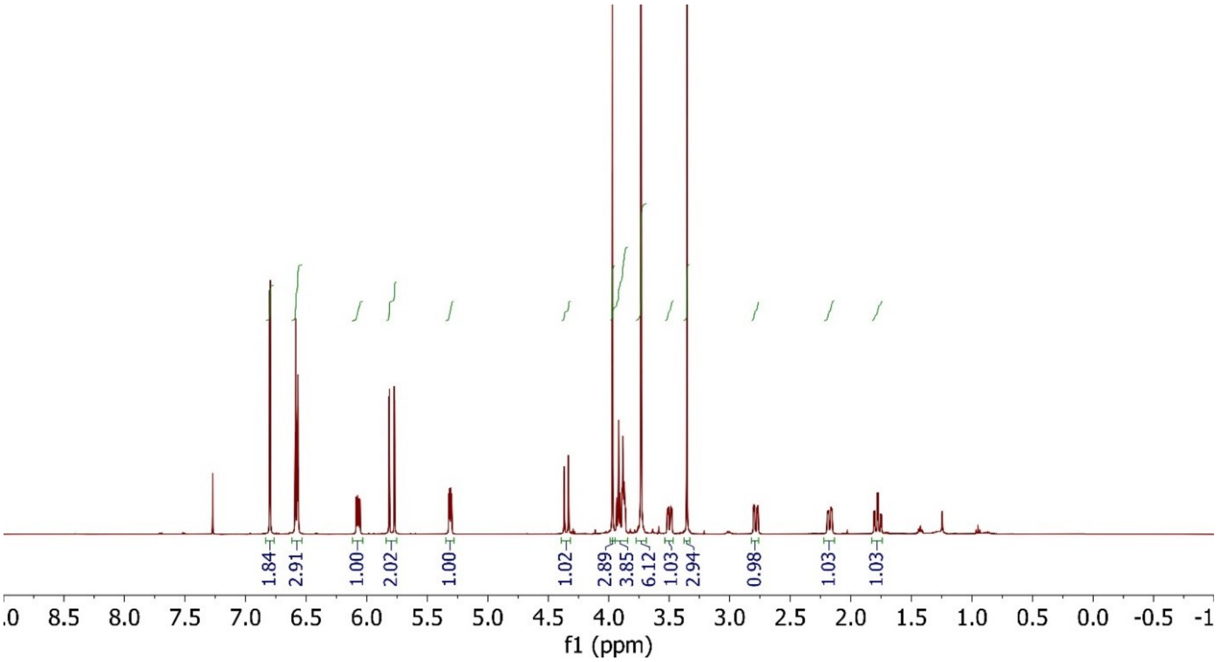

Figure S5. ^{1}H NMR spectrum of 11-*O*-(3,5-dimethoxybenzoyl)ambelline (**28m**)

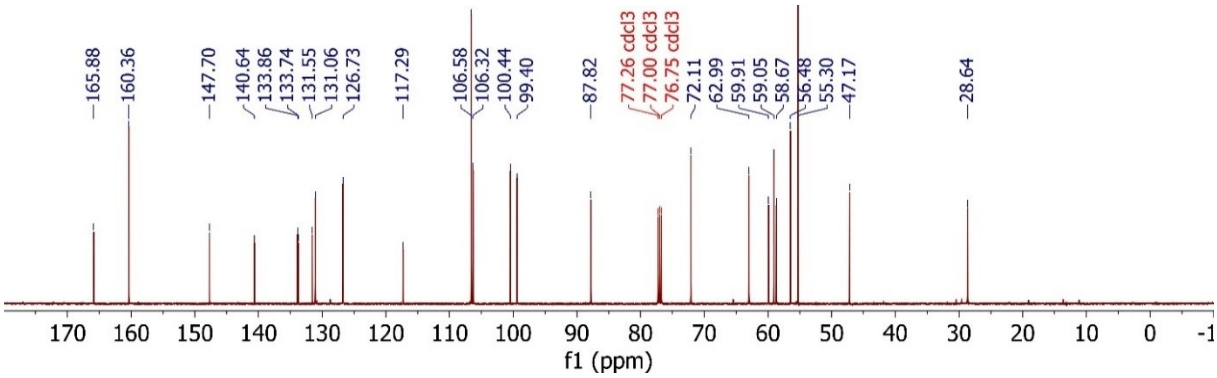

Figure S6. ^{13}C NMR spectrum of 11-*O*-(3,5-dimethoxybenzoyl)ambelline (**28m**)

*3) 11-O-(3,4,5-Trimethoxybenzoyl)ambelline (**28n**)*

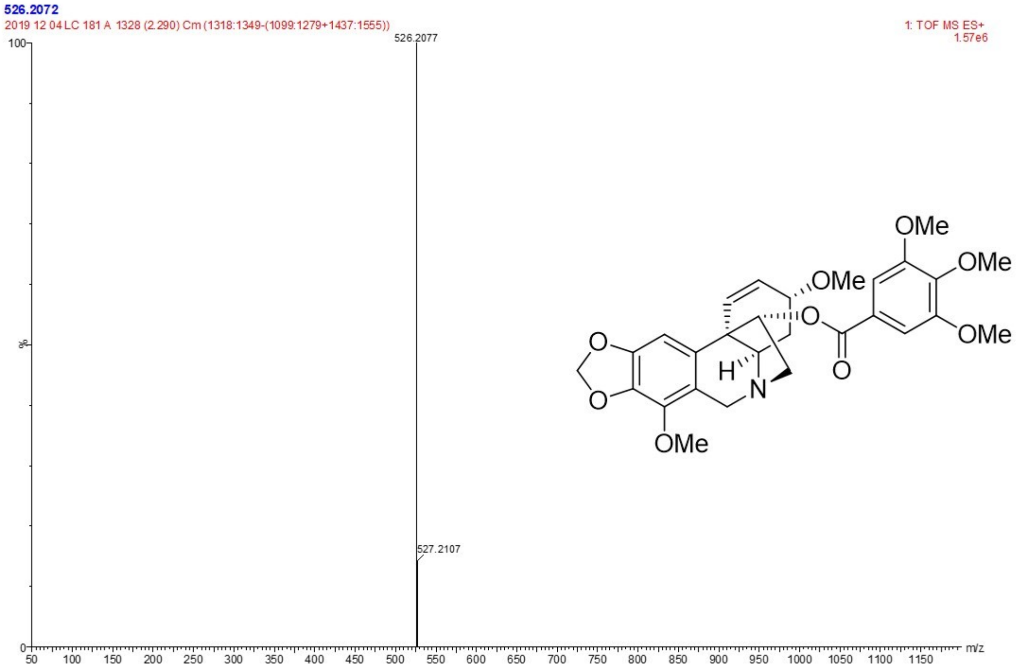

Figure S7. ESI-HRMS spectrum of 11-*O*-(3,4,5-trimethoxybenzoyl)ambelline (**28n**)

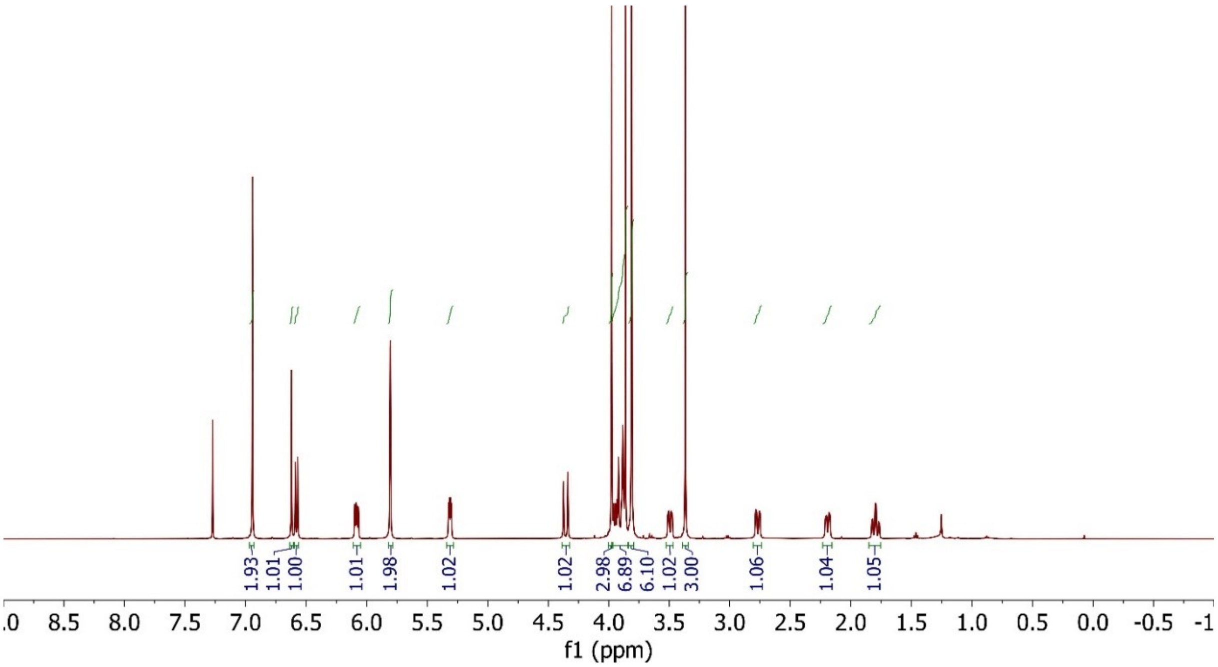

Figure S8. ^{1}H NMR spectrum of 11-*O*-(3,4,5-trimethoxybenzoyl)ambelline (**28n**)

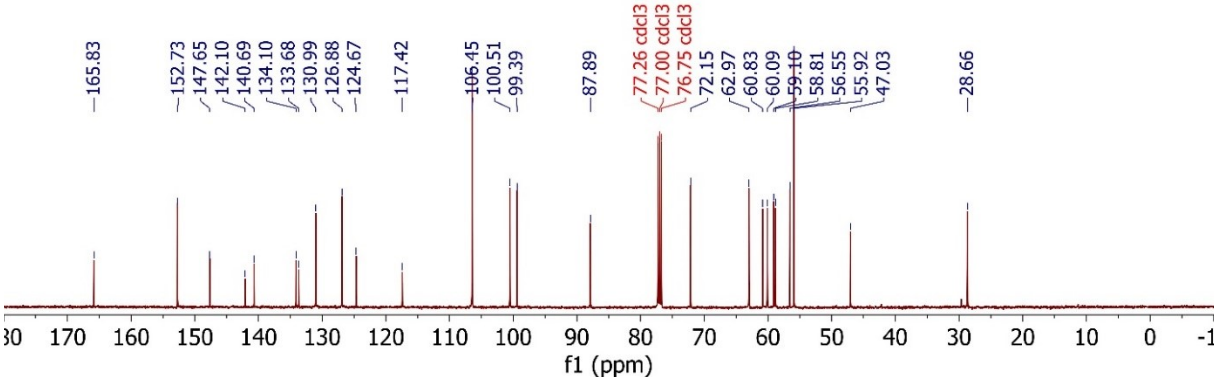

Figure S9. ^{13}C NMR spectrum of 11-*O*-(3,4,5-trimethoxybenzoyl)ambelline (**28n**)

4) 11-O-(4-Methyl-3-nitrobenzoyl)ambelline (28r)

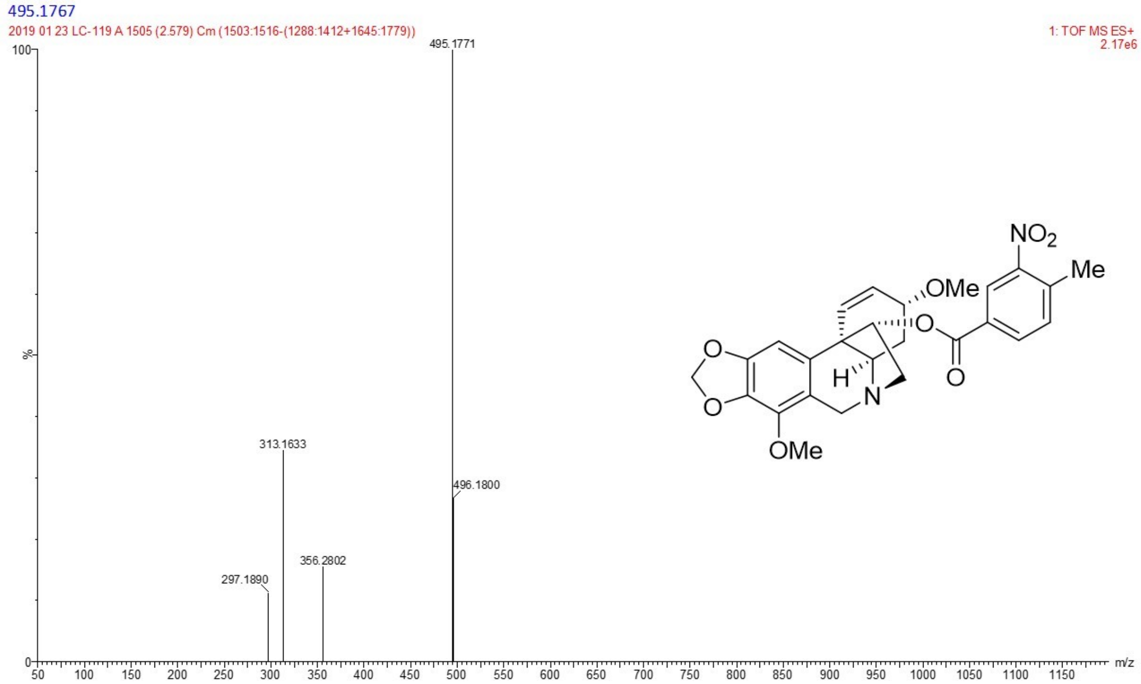

Figure S10. ESI-HRMS spectrum of 11-*O*-(4-methyl-3-nitrobenzoyl)ambelline (**28r**)

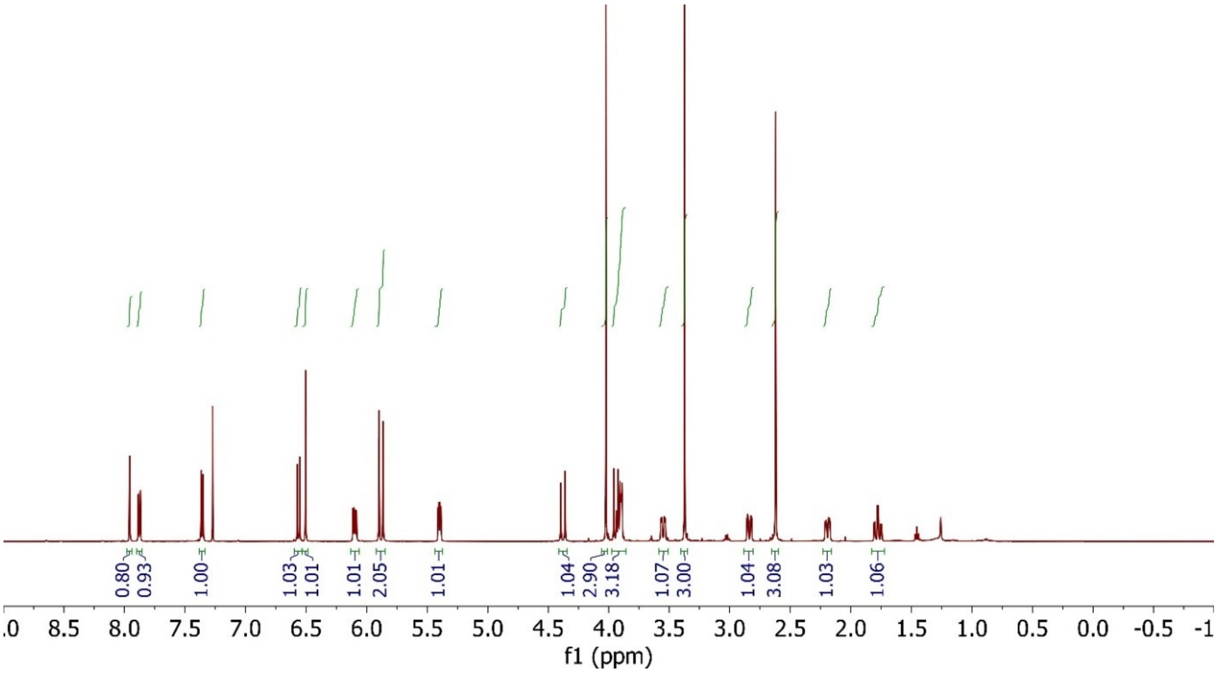

Figure S11. ^{1}H NMR spectrum of 11-*O*-(4-methyl-3-nitrobenzoyl)ambelline (**28r**)

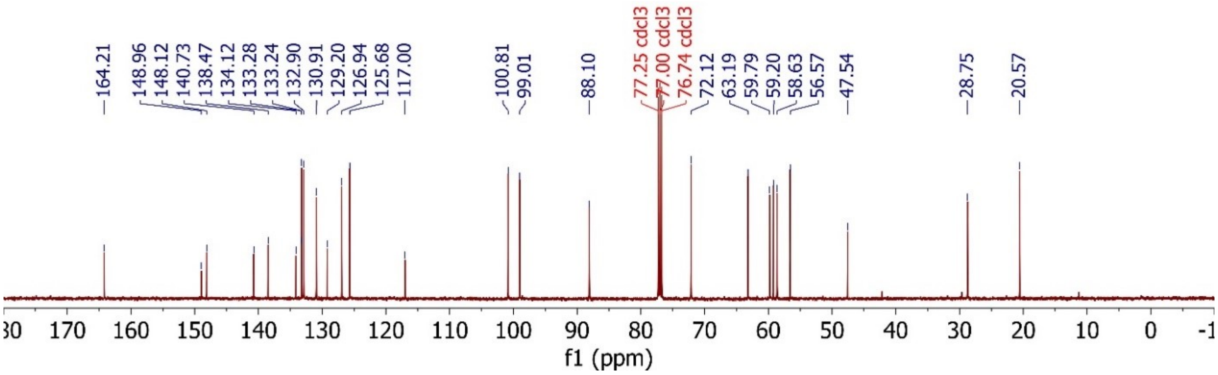

Figure S12. ^{13}C NMR spectrum of 11-*O*-(4-methyl-3-nitrobenzoyl)ambelline (**28r**)

*5) 11-O-(2-Chloro-4-nitrobenzoyl)ambelline (**28s**)*

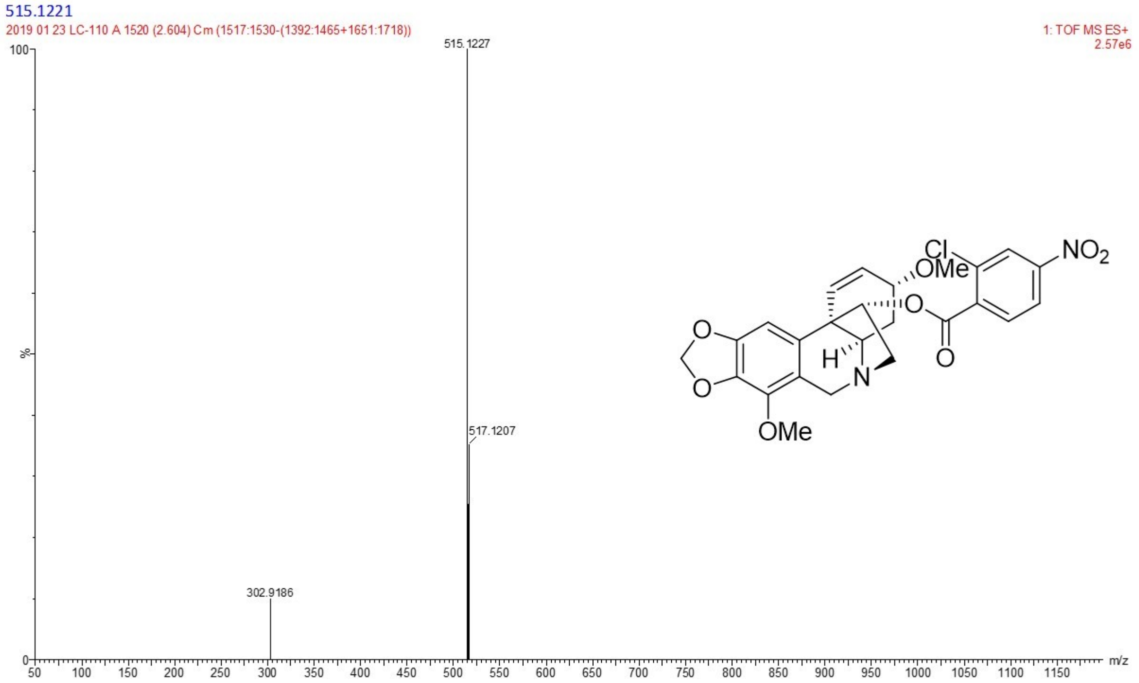

Figure S13. ESI-HRMS spectrum of 11-*O*-(2-chloro-4-nitrobenzoyl)ambelline (**28s**)

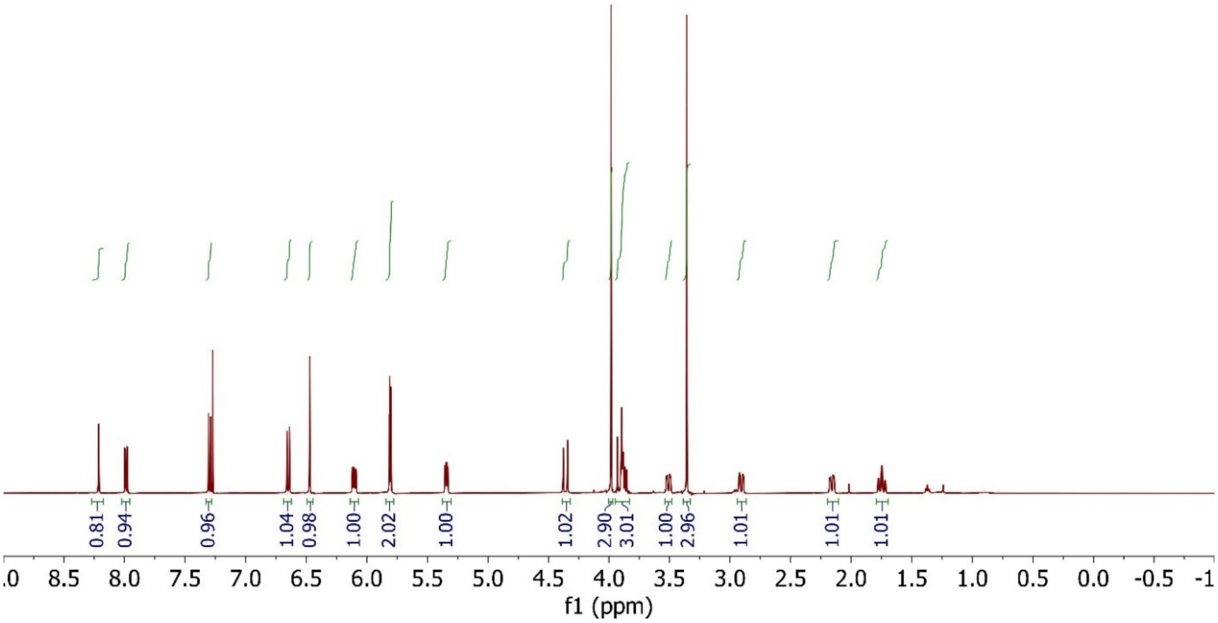

Figure S14. ¹H NMR spectrum of 11-*O*-(2-chloro-4-nitrobenzoyl)ambelline (**28s**)

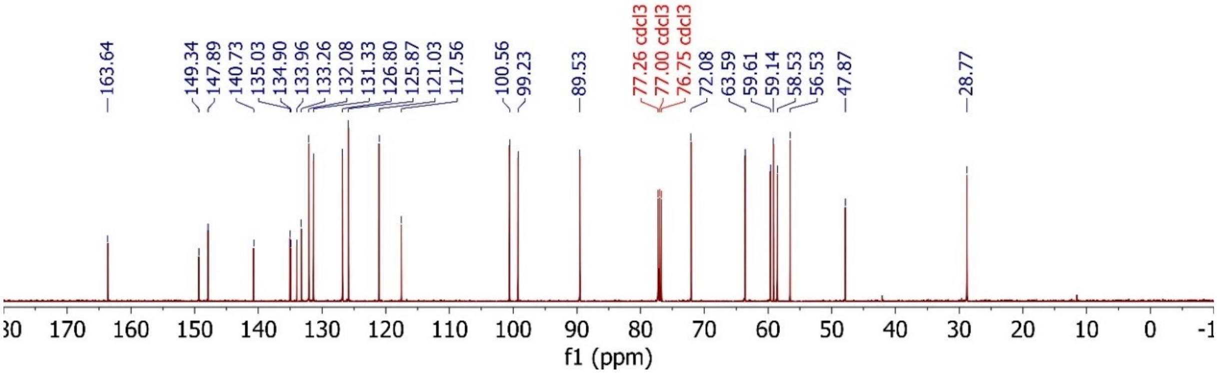

Figure S15. ^{13}C NMR spectrum of 11-*O*-(2-chloro-4-nitrobenzoyl)ambelline (**28s**)

*6) 11-O-(4-Chloro-3-nitrobenzoyl)ambelline (**28t**)*

ESI-HRMS *m/z* calcd for $C_{25}H_{23}ClN_2O_8$ $[M+H]^+$ 515.1216 found 515.1224.

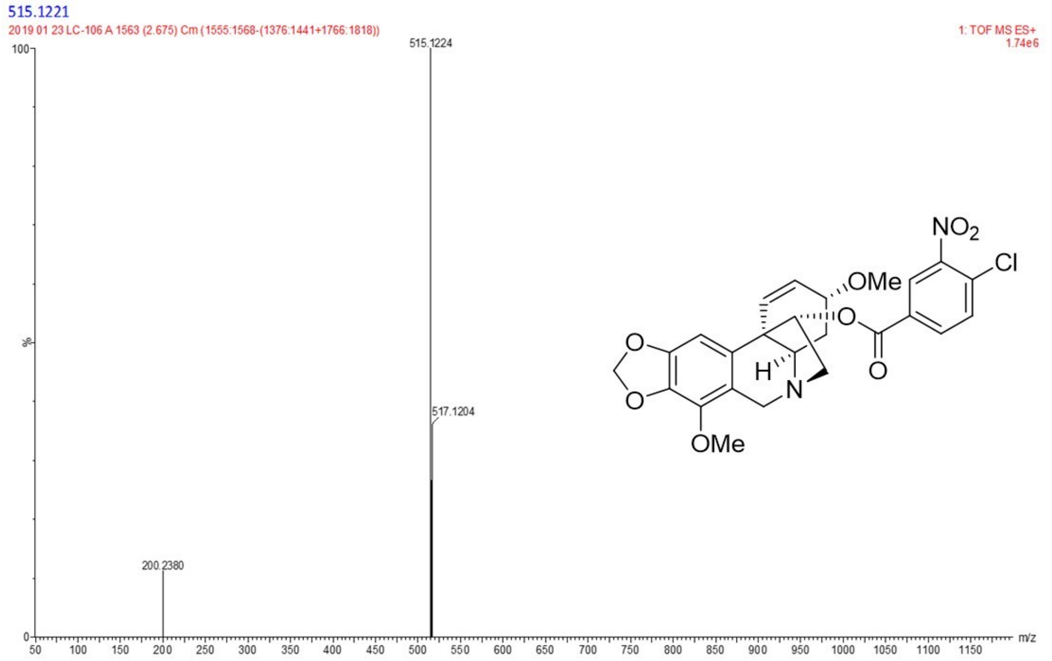

Figure S16. ESI-HRMS spectrum of 11-*O*-(4-chloro-3-nitrobenzoyl)ambelline (**28t**)

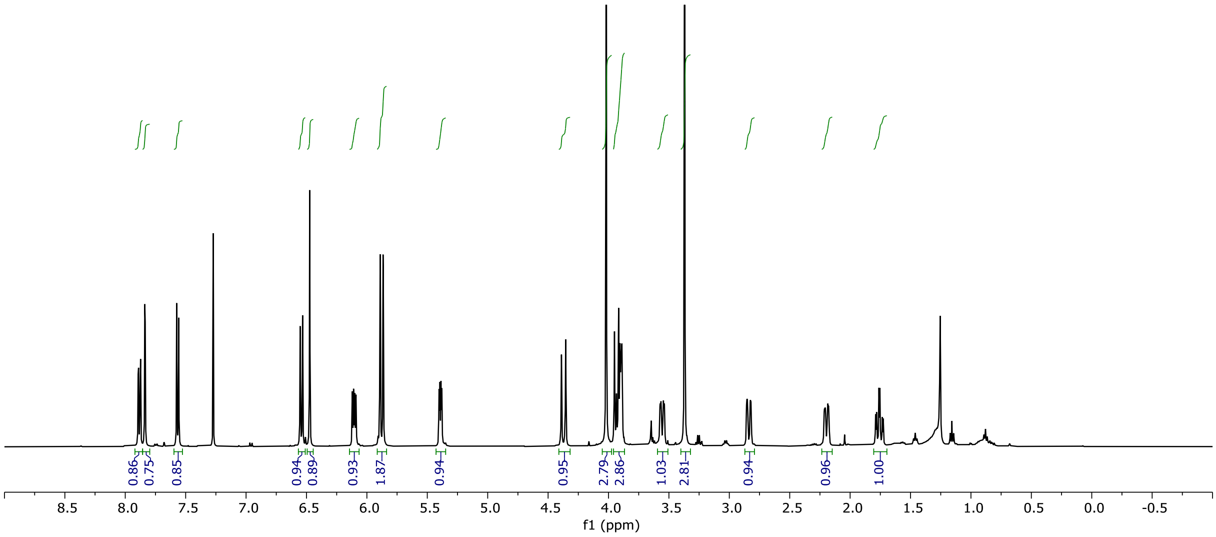

Figure S17. ^{1}H NMR spectrum of 11-*O*-(4-chloro-3-nitrobenzoyl)ambelline (**28t**)

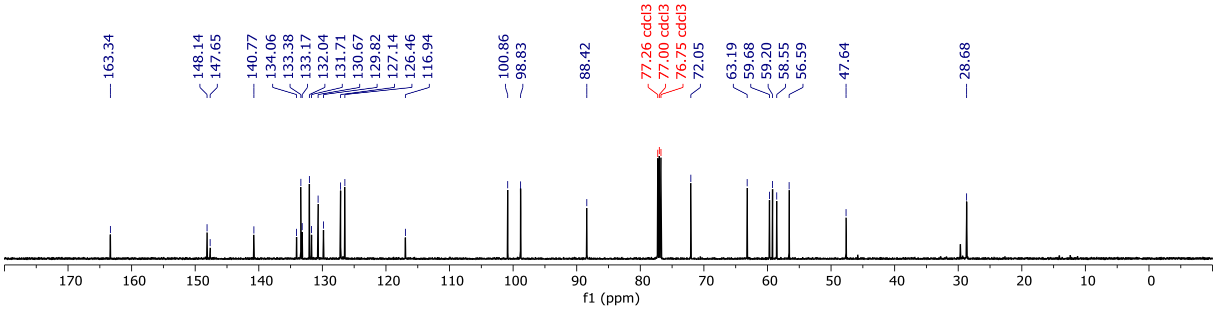

Figure S18. ^{13}C NMR spectrum of 11-*O*-(4-chloro-3-nitrobenzoyl)ambelline (**28t**)
